# Brain tight junction protein expression in sepsis in an autopsy series

**DOI:** 10.1186/s13054-020-03101-3

**Published:** 2020-06-29

**Authors:** Kristo Erikson, Hannu Tuominen, Merja Vakkala, Janne Henrik Liisanantti, Tuomo Karttunen, Hannu Syrjälä, Tero Ilmari Ala-Kokko

**Affiliations:** 1 Division of Intensive Care Medicine, Department of Anesthesiology, Research Group of Surgery, Anesthesiology and Intensive Care Medicine, Oulu University Hospital, Medical Research Center Oulu, University of Oulu, Oulu, Finland; 2 Department of Anesthesiology, Intensive Care Center, North Estonia Medical Centre, Tallinn, Estonia; 3 Department of Pathology and Department of Infection Control, Oulu University Hospital, Medical Research Center Oulu, University of Oulu, Oulu, Finland

**Keywords:** Sepsis, Tight junction protein expression, BBB damage

## Abstract

**Background:**

Neuroinflammation often develops in sepsis along with increasing permeability of the blood-brain barrier (BBB), which leads to septic encephalopathy. The barrier is formed by tight junction structures between the cerebral endothelial cells. We investigated the expression of tight junction proteins related to endothelial permeability in brain autopsy specimens in critically ill patients deceased with sepsis and analyzed the relationship of BBB damage with measures of systemic inflammation and systemic organ dysfunction.

**Methods:**

The case series included all (385) adult patients deceased due to sepsis in the years 2007–2015 with available brain specimens taken at autopsy. Specimens were categorized according to anatomical location (cerebrum, cerebellum). The immunohistochemical stainings were performed for occludin, ZO-1, and claudin. Patients were categorized as having BBB damage if there was no expression of occludin in the endothelium of cerebral microvessels.

**Results:**

Brain tissue samples were available in 47 autopsies, of which 38% (18/47) had no expression of occludin in the endothelium of cerebral microvessels, 34% (16/47) developed multiple organ failure before death, and 74.5% (35/47) had septic shock.

The deceased with BBB damage had higher maximum SOFA scores (16 vs. 14, *p* = 0.04) and more often had procalcitonin levels above 10 μg/L (56% vs. 28%, *p* = 0.045) during their ICU stay. BBB damage in the cerebellum was more common in cases with C-reactive protein (CRP) above 100 mg/L as compared with CRP less than 100 (69% vs. 25%, *p* = 0.025).

**Conclusions:**

In fatal sepsis, damaged BBB defined as a loss of cerebral endothelial expression of occludin is related with severe organ dysfunction and systemic inflammation.

## Introduction

Sepsis is a life-threatening organ dysfunction caused by a dysregulated host response to an infection [1]. It is a common cause of death among critically ill patients. Sepsis is associated with widespread endothelial cell damage from uncontrolled inflammation and leads to multiorgan failure. It has been suggested that neuroinflammation develops in sepsis along with an activation of the cerebral endothelium, an increase in the permeability of the blood-brain barrier (BBB) and a promotion of neutrophil infiltration; all these abnormalities result in brain dysfunction [2, 3]. Subsequent septic encephalopathy is associated with a further breakdown of the blood-brain barrier [4] and manifests clinically as an altered mental status and disturbed consciousness, and leads to increased mortality as well as long-term cognitive dysfunction in survivors [5].

The blood-brain barrier (BBB) is a structural and biochemical barrier that regulates the entry of molecules from the plasma into the brain and preserves ionic homeostasis within the brain [6]. The functionally important part of the barrier is formed by tight junction (TJ) structures between the endothelial cells. TJs are composed of—among other proteins—occludin, claudin-5, and ZO-1, which are all present in human brain endothelial cells [7]. Several endogenous and exogenous substances, such as proinflammatory cytokines, reactive oxygen species, and bacterial toxins increase the permeability of TJs. Most of the studies thus far have been based on animal data and in vitro cell culture methods [8, 9], and there are only a limited number of human studies on BBB dysfunction in sepsis and association of the dysfunction with clinical features such as systemic level inflammation. Previous clinical research on the pathophysiology of septic encephalopathy has revealed cytotoxic or vasogenic edema as the most consistently reported MRI change in septic encephalopathy [10, 11]. Postmortem human studies have revealed mainly ischemic lesions and diffuse neuroaxonal injury [12], and—in a limited series of three cases—chemokine and cytokine expression has been shown to be upregulated [13].

To gain more insight into the pathophysiology of brain dysfunction in human sepsis, we investigated the expression of proteins related to endothelial permeability in brain autopsy specimens in critically ill patients deceased with sepsis. Our hypothesis was that sepsis induces downregulation of tight junction proteins leading to BBB damage and that alterations in these proteins are related to the severity of sepsis and systemic inflammation.

## Material and methods

### Patients

The study was approved by the ethics committee of the Oulu University Hospital and the Northern Ostrobothnia Hospital District (224/2013). The use of brain tissue samples obtained in the autopsy was approved by the National Institute for Health and Welfare (BB_2017_1003). This retrospective observational cohort study was conducted at the Oulu University Hospital, Oulu, Finland, an academic tertiary level referral hospital. Adult patients deceased with sepsis who underwent postmortem examination during the years 2007–2015 and had brain tissue specimens available were included in the study. Medical non-forensic autopsies are performed if antemortem data or clinical examination does not provide the clinician enough information to issue a death certificate and to better understand the disease course, or in cases in which the next of kin request it. The final decision is made at the discretion of the treating physician. All patients were treated by a multidisciplinary team of intensivists and infectious disease specialists in our 26 beds, closed adult intensive care unit. Sepsis was defined according to the American College of Chest Physicians/Society of Critical care medicine criteria [14]. The intensive care treatment was performed according to normal intensive care unit (ICU) protocols and sepsis guidelines.

### Clinical data collection

Clinical parameters were gathered from the ICU clinical data management system database (Centricity Critical Care, Clinisoft; GE Healthcare, Helsinki, Finland). On admission, the severity of illness was determined by the Acute Physiology and Chronic Health (APACHE II) score [15], and daily total and individual organ group sequential organ failure assessment score (SOFA) was used as a measure of organ dysfunction [16]. The Glasgow Coma Score (GCS) was employed for assessing the impairment of patients’ level of consciousness [17]. The presence of multi-organ failure (MOF) during the ICU treatment was defined as more than two organs failing based on grade 3 or 4 sequential organ failure assessment [16]. Data regarding age, sex, time to death, focus of infection, and blood culture positivity were collected. Procalcitonin (PCT) on admission and its highest value, as well as white blood cell count were retrieved. Clinical laboratory samples were analyzed by commercially available laboratory methods in the hospital accredited central laboratory (NordLab, Oulu University Hospital, Oulu, Finland).

### Immunohistochemical stainings

The median time from the death to autopsy was 5 (3–6) days. At the autopsy, brain specimens were routinely collected, usually from a least two anatomical locations. A systematic neuropathological examination was performed when the pathologist in charge considered it necessary or by request of the clinician, for example, in cases when the patient had neurological symptoms. For each deceased subject, brain tissue specimens were categorized according to anatomical location (cerebrum, cerebellum), and one representative tissue block from each location was selected for the study. The exact location of the specimens had not been registered in all cases, but according to the suggested sampling routine of our hospital, likely followed in most cases, cerebral samples represented the precentral gyrus, and the cerebellar samples the lateral aspect of either hemisphere. The immunohistochemical stainings were performed in accordance with the manufacturer’s recommendation, and a set of samples was used to optimize the dilution of primary antibodies. We used the following antibodies and conditions: (1) human occludin (rabbit polyclonal, catalog no: 711500, Invitrogen, Frederick, MD, USA), with pretreatment using pronase, at a dilution of 1:800, incubation at room temperature (RT) 60 min; (2) ZO-1 (rabbit polyclonal; Zymed, cat no: 61-7300, Carlsbad CA, USA), pretreatment with 15 min boiling in a TRIS-EDTA buffer, dilution at 1:400, incubation for 60 min at RT); and (3) claudin-5 (mouse monoclonal, clone 4C3C2, Zymed), pretreatment with TRIS-EDTA, dilution at 1:50, incubation for 60 min at RT. For all antibodies, the detection was performed with a polymer-based kit (Envision, Dako, Copenhagen, Denmark). Diaminobenzidine (Dako basic DAB-kit, Dako) was used as a chromogen. All stainings were performed with the Dako Autostainer (Dako, Copenhagen, Denmark). Validation of our immunohistochemical analysis was performed through a series of negative controls by omitting the primary antibody.

### Evaluation of immunohistochemical stainings

Stained sections were digitized (Leica-Aperio AT2; Leica Biosystems, Nussloch, Germany) and assessed using the Aperio ImageScope program independently by two researchers (KE and ME) supervised by an experienced neuropathologist (HT), all blinded for the clinical and outcome data. Endothelial cells of the capillaries were analyzed for each anatomic location. Expression was considered positive if 50% or more showed a positive reaction of any degree and were negative in the absence of any staining or if less than 50% showed a positive reaction.

### Detection of BBB dysfunction

Occludin is the key tight junction (TJ) protein in cerebral endothelial cells which modulates blood-brain barrier (BBB) functions and maintains TJ stability, and accordingly, patients were categorized as having BBB damage if there was no expression of occludin in the endothelium of the cerebral microvessels [18, 19].

### Outcome variables

The associations of an absence of occludin to the severity of illness were evaluated by comparison with the maximum SOFA score recorded during the ICU stay, an increase of PCT to > 10 μg/L and CRP to > 100 mg/L. The cut-off point for CRP as a marker of inflammation was chosen according to Falk et al. [20]. The cutoff value for PCT > 10 μg/L was used as a marker of organ dysfunction in sepsis [21].

### Statistical analysis

Statistical analyses were performed with SPSS for Windows (2017 release, Version 25; IBM Corporation, Armonk, NY, USA). Data are expressed as the percentage of stained cells as median (25–75th percentile) in continuous variables. Categorical data were analyzed with Fisher’s exact test. Mann-Whitney *U* test was applied to distributions across the two groups. Spearman’s correlation coefficient (rho) was calculated. Two-sided *p* values are presented and a *p* < 0.05 was considered statistically significant. However, the level of statistical significance should be treated with caution, given the large number of statistical tests performed.

## Results

**Fig. 1:**
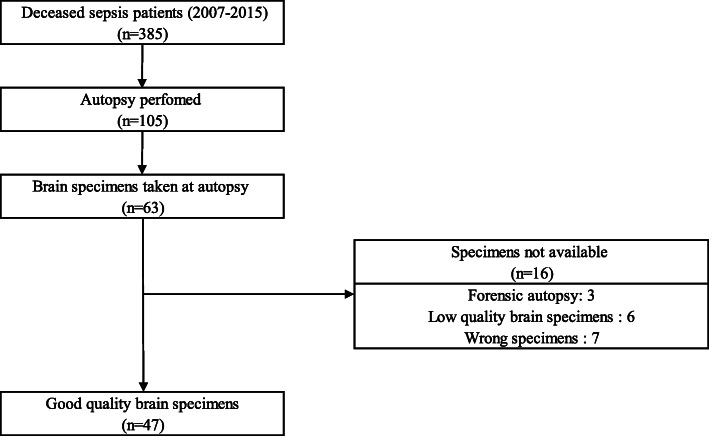
Patients inclusion and exclusion criteria

During the study period from 2007 to 2015, 105 patients deceased due to sepsis had autopsies. Brain tissue samples were available in 47 autopsies (Fig. 1). Specimens from the cerebrum were available in 47 subjects (100%), and from the cerebellum in 32 subjects (68%). The main macroscopic pathological findings were cerebral oedema (8.5%), meningioma (2%), cerebral infarctions (4.2%), intracranial hemorrhages (4.2%), cerebral arteriosclerosis (6.3%), leukemic lesion (2%), and encephalitic oedema (2%).

All other macroscopic examples were normal. The median age on admission was 63 years (56–69), 34% (16/47) developed multiple organ failure before death. All patients with MOF also had septic shock. Seventy-five percent (35/47) had a septic shock while 26 (55%) received corticosteroids for vasopressor-resistant shock. The median time to death was 1.2 days (0.6–6.9) (Table 1).

**Table 1:** Demographic data and clinical features

Parameter	All, *N* = 47	BBB damage, *N* = 18	No BBB damage, *N* = 29	*p*
Age, years, median (percentiles)	62.5 (56–69)	61 (56–69)	63 (60–69.50)	0.7
Sex (male/female)	34/13	14/4	20/9	0.73
APACHE II, admission, median (percentiles)	24 (18–30)	27 (22–31)	24 (17–29)	0.16
SOFA score, admission, median (percentiles)	11 (7–14)	11 (8–15)	11 (7–14)	0.47
SOFA score, maximum, median (percentiles)	15 (12–17)	16.5 (15–20)	14 (11–15.5)	**0.04**
GCS admission, median (percentiles)	15 (6–15)	15 (8–15)	14 (6–15)	0.8
GCS less than 13, *n* (%)	22 (47%)	9 (50%)	12 (42%)	0.48
Operated, *n* (%)	10 (21.3%)	2 (11.1%)	8 (27.6%)	0.27
Intra-abdominal surgery, *n* (%)	7 (14.9%)	2 (11.1%)	5 (17.2%)	0.7
LOS (days), median (percentiles)	1.2 (0.6–6.9)	2.3 (0.96–9.14)	0.79 (0.4–6.72)	0.228
Septic shock, *n* (%)	35 (74.5%)	14 (78%)	21 (75%)	0.74
Multiple organ failure, *n* (%)	16 (34%)	6 (12.7%)	10 (55.6%)	0.9
Received MV, *n* (%)	41 (88%)	17 (95%)	24 (83%)	0.384
Received ARRT, *n* (%)	25 (53%)	13 (72%)	12 (42%)	0.07

*P value calculated using Fisher’s exact test

*APACHE II* Acute Physiology and Chronic Health Evaluation II, *ARRT* acute renal replacement therapy, *ICU* intensive care unit, *LOS* length of stay, *SAPS II* Simplified Acute Physiology Score, *SOFA* Sequential Organ Failure Assessment score, *MV* mechanical ventilation, *GCS* Glasgow Coma Scale

Of the entire group of patients, 22 (47%) had a positive blood culture; 13 (28%) with a gram-positive bacteria (*Streptococcus pneumoniae, n* = 3*; Staph aureus, n* = 2*; Streptococcus betahemolyticus, n* = 3; *Coagulase negative staphylococci, n* = 3; *Enterococcus, n* = 2), and 9 (19%) with gram-negative bacteria (*E. coli, n = 3; Klebsiella pneumoniea, n = 2; Pseudomonas aeroginosa, n = 2; Enterobacter aerogenes, n = 1; Capnocytophaga canimorsus, n = 1*). In addition, one patient had an anaerobic bacterium (*Clostridium septicum*) (Table 2). Antimicrobial treatment was directed according to microbiological findings. The foci of infections were as follows: lungs 40%, abdomen 23%, soft tissue 6%, urinary 2%, and primary septichemia 28%. Two of the patients (4%) also had a central nervous system infection (*Streptococcus pneumoniae, n = 1, Capnocytophaga canimorsus, n = 1).* There were no differences in the frequencies of the foci of infections between those with BBB damage and those without.

**Table 2:** Microbiology and systemic inflammation markers

Parameter	All, *N* = 47	BBB damage, *N* = 18	No BBB damage, *N* = 29	*p*
The highest PCT > 10 on stay, *n* (%)	18 (38.3%)	10 (56%)	8 (27.6%)	**0.045**
The highest CRP > 100 on stay, *n* (%)	23 (49%)	10 (56%)	13 (45%)	0.47
CRP on admission, median (percentiles)	104 (34–161)	105.5 (35–154)	76 (26–206)	0.9
PCT on admission, median (percentiles)	1625 (0.28–28.5)	3.3 (1.3–35.9)	0.8 (0–20.4)	0.7
Leuc on admission, median (percentiles)	11.1 (3.5–15.3)	14.1 (6.3–19.2)	10 (3.5–15)	0.23
Blood culture positive, *n* (%)	22 (47%)	9 (50%)	13 (45%)	0.7
Gram positive, *n* (%)	13 (28%)	5 (28%)	8 (28%)	0.7
Gram negative, *n* (%)	9 (19%)	4 (22%)	5 (17%)	0.7
Received corticosteroid, *n* (%)	26 (55%)	9 (50%)	17 (58.6%)	0.53

*P value calculated using Fisher’s exact test

*CRP* C-reactive protein, *PCT* procalcitonin, *Leuc* leukocytes

**Fig. 2:**
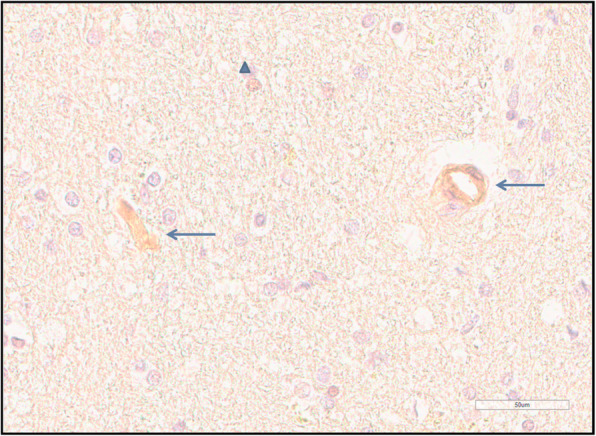
Occludin immunohistochemistry shows a positive reaction in capillary endothelium (arrow) and in a glial cell nucleus (triangle)

**Fig. 3:**
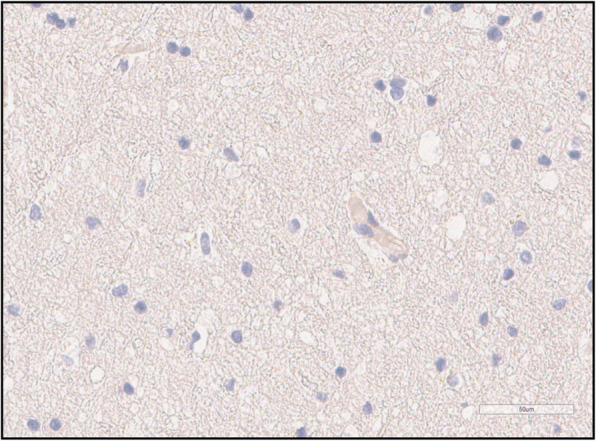
Occludin immunohistochemistry shows no reaction in capillary endothelium and glial nuclei

A systematic evaluation of the tight junction protein expression in brain samples indicated that there was a distinct variation between the cases (Table 3). Occludin expression (Fig. 2) in the endothelium of the cerebral microvessels was missing in 38% (18/47) of samples. Positive ZO-1 staining was practically absent from the endothelial cells (Fig. 3). Claudin-5 was similarly absent from the endothelium. Cerebrum endothelial occludin expression was present in 62% of cases, and in the cerebellum in 32% of cases.

**Table 3:** Absence (% of cases) of claudin-5 and ZO-1 expression in different brain anatomical area and presence or absence of BBB damage

Area	All, *N* = 47	BBB damage, *N* = 18	No BBB damage, *N* = 29	*p*
**Cerebrum**				
**ZO1**				
Endothelium	1 (2.1%)	1 (5.6%)	0 (0%)	0.383
**Claudin-5**				
Endothelium	2 (4.3%)	1 (5.6%)	1 (3.5%)	> 0.9
**Cerebellum**				
**ZO1**				
Endothelium	0 (0%)	0 (0%)	0 (0%)	NA
**Claudin-5**				
Endothelium	0 (0%)	0 (0%)	0 (0%)	NA

After our systematic evaluation, we assessed the relationship between tight junction protein expression and clinical findings. A high blood CRP (> 100 mg/L) was associated with an absence of occludin expression in cerebellar endothelium as compared to low CRP (< 100 mg/L; 69% vs. 25%; *p* = 0.025). Similarly, high blood PCT (> 10 μg/L) was associated with an absence of occludin expression in cerebral endothelial cells as compared to lower PCT (< 10 μg/L; 56% vs 26%, *p* = 0.045).

In cases with BBB damage (absent occludin in cerebral endothelium), the maximum SOFA score and percentage of patients with PCT levels above 10 μg/L were higher than in those without BBB damage (Table 2).

## Discussion

There is experimental evidence that damage to the BBB involving intercellular junctions of the endothelial cells is an essential stage in the development of septic encephalopathy, but there is only scanty and indirect human data supporting this concept. To our knowledge, this is the first study describing immunohistochemical findings of TJ expression in clinical human autopsy specimens in sepsis. Interestingly, the high organ failure scores and biomarkers of systemic inflammation were associated with a loss of TJ protein expression. These findings support the role of BBB damage involving TJs in human sepsis. Our results bring new insight to the pathophysiology of sepsis-related brain dysfunction, highlighting the occurrence of variable responses in different brain regions and different types of cells and levels of markers of the severity of illness.

In our series, in cases with a CRP level above 100 mg/L, the proportion of occludin-negative samples was higher in the cerebellar endothelium. It has recently been presented that the neuroinflammatory cascade has been strongly linked to elevated levels of pro-inflammatory cytokines which can lead to dysregulated BBB permeability [22, 23]. According to mice studies, one of the main mechanisms is the proinflammatory status, which causes the activation and dysfunction of cerebrovascular endothelial cells [23, 24]. Proinflammatory cytokines are important mediators for inducing the C-reactive protein response [25].

Occludin is a key tight junction protein in cerebral endothelial cells and plays an important role in modulating blood-brain barrier (BBB) functions. In our series, the absence of occludin in cerebral microvascular endothelia was related to a more severe disease and a higher level in the inflammatory response. These findings are in good agreement with findings from rodent studies. In a rat model, cerebral ischemia induced an increase of blood occludin with a loss of occludin from ischemic cerebral microvessels and BBB damage [26]. Furthermore, in an in vitro human cerebral endothelial cell model, pro-inflammatory cytokines and endotoxin resulted in a significant decrease in the expression of occludin [27]. Similarly, human cerebral microvascular endothelial cells in vitro exposed to endotoxin decreased levels of occludin and ZO-1 [28].

In our series, the expression of claudin 5 was only present in 4% of the cerebral endothelial samples. In an in vitro study, the endotoxin exposure of human brain microvascular endothelial cells increased the permeability of the BBB with lowered expression levels of occludin and claudin-5 [29].

### Clinical significance

Our study stresses the importance of BBB damage in sepsis. The results of the present study support those reported in experimental studies, but also new findings of clinical associations were found. Our findings suggest that analyzing of blood occludin concentration might serve as a biomarker in human sepsis for the detection of BBB damage, as already shown in a rat study with cerebral arterial occlusion [30]. We have earlier shown that delirium in septic shock was associated with an elevation in protein S-100β expression [31]. Modulating BBB function may also offer new treatment possibilities for preventing brain dysfunction. For example, an in vitro model of human BBB composed of brain microvascular endothelial cells showed that anti-TNF-α antibody improved the upregulation of tight junction protein expressions following oxidative stress [32].

### Limitations

The collection of multiple brain samples was not standardized as the autopsies were performed when judged to be clinically pertinent. It is possible that the autopsies were only performed in the deceased with whom there were diagnostic difficulties during their ICU stay. Moreover, at the time of their death, the patients were at different stages of inflammation. Due to the retrospective study design, we were not able to include focused data on the brain function during the ICU stay including EEG or MRI of the brain. Further studies with comparison to non-septic patients are needed to explore the link between sepsis and TJ proteins.

## Conclusions

Patients with damaged BBB defined as a missing cerebral endothelial expression of occludin developed more severe organ dysfunctions and more severe inflammation.

## Data Availability

All data generated or analyzed during this study are included in this published article.

## Abbreviations

APACHE II: Acute Physiology and Chronic Health Evaluation II
ARRT: Acute renal replacement therapy
BBB: Blood-brain barrier
CRP: C-reactive protein
GCS: Glasgow Coma Scale
ICU: Intensive care unit
Leuc: Leukocytes
LOS: Length of stay
MV: Mechanical ventilation
PCT: Procalcitonin
SAPS II: Simplified Acute Physiology Score
SOFA: Sequential Organ Failure Assessment
TJ: Tight junction

## References

[1] Singer M, Deutschman CS, Seymour CW, Shankar-Hari M, Annane D, Bauer M, Bellomo R, Bernard GR, Chiche J, Coopersmith CM, Hotchkiss RS, Levy MM, Marshall JC, Martin GS, Opal SM, Rubenfeld GD, van der Poll T, Vincent J, Angus DC (2016). The third international consensus definitions for sepsis and septic shock (sepsis-3). JAMA.

[2] McGrane S, Girard TD, Thompson JL, Shintani AK, Woodworth A, Ely EW, Pandharipande PP (2011). Procalcitonin and C-reactive protein levels at admission as predictors of duration of acute brain dysfunction in critically ill patients. Critical care (London, England).

[3] Ziaja M (2013). Septic encephalopathy. Curr Neurol Neurosci Rep.

[4] Davies DC (2002). Blood–brain barrier breakdown in septic encephalopathy and brain tumours. J Anat.

[5] Pandharipande PP, Girard TD, Jackson JC, Morandi A, Thompson JL, Pun BT, Brummel NE, Hughes CG, Vasilevskis EE, Shintani AK, Moons KG, Geevarghese SK, Canonico A, Hopkins RO, Bernard GR, Dittus RS, Ely EW (2013). Long-term cognitive impairment after critical illness. N Engl J Med.

[6] Abbott NJ, Patabendige AAK, Dolman DEM, Yusof SR, Begley DJ (2009). Structure and function of the blood–brain barrier. Neurobiol Dis.

[7] Tietz S, Engelhardt B (2015). Brain barriers: crosstalk between complex tight junctions and adherens junctions. J Cell Biol.

[8] Taccone FS, Su F, Pierrakos C, He X, James S, Dewitte O, Vincent J, De Backer D (2010). Cerebral microcirculation is impaired during sepsis: an experimental study. Critical Care (London, England).

[9] du Moulin GC, Paterson D, Hedley-Whyte J, Broitman SA (1985). E. coli peritonitis and bacteremia cause increased blood-brain barrier permeability. Brain Res.

[10] Stubbs DJ, Yamamoto AK, Menon DK (2013). Imaging in sepsis-associated encephalopathy—insights and opportunities. Nat Rev Neurol.

[11] Piazza O, Cotena S, De Robertis E, Caranci F, Tufano R (2009). Sepsis associated encephalopathy studied by MRI and cerebral spinal fluid S100B measurement. Neurochem Res.

[12] Ehler J, Barrett LK, Taylor V, Groves M, Scaravilli F, Wittstock M, Kolbaske S, Grossmann A, Henschel J, Gloger M, Sharshar T, Chretien F, Gray F, Nöldge-Schomburg G, Singer M, Sauer M, Petzold A (2017). Translational evidence for two distinct patterns of neuroaxonal injury in sepsis: a longitudinal, prospective translational study. Critical Care (London, England).

[13] Warford J, Lamport A, Kennedy B, Easton AS: Human brain chemokine and cytokine expression in sepsis: a report of three cases*.* The Canadian journal of neurological sciences J Can Sci Neurol 2017, 44(1):96–104. 10.1017/cjn.2016.310 27832827

[14] Anonymous American College of Chest Physicians/Society of Critical Care Medicine Consensus Conference (1992). Definitions for sepsis and organ failure and guidelines for the use of innovative therapies in sepsis. Critical Care Med.

[15] Knaus WA, Draper EA, Wagner DP, Zimmerman JE (1985). APACHE II: a severity of disease classification system. Crit Care Med.

[16] Vincent J, de Mendonca A, Cantraine F, Moreno R, Takala J, Suter PM, Sprung CL, Colardyn F, Blecher S (1998). Use of the SOFA score to assess the incidence of organ dysfunction/failure in intensive care units: results of a multicenter, prospective study. Crit Care Med.

[17] Bastos P, Sun X, Wagner D, Wu A, Knaus W (1993). Glasgow coma scale score in the evaluation of outcome in the intensive care unit: findings from the acute physiology and chronic health evaluation III study. Crit Care Med.

[18] Hawkins BT, Davis TP (2005). The blood-brain barrier/neurovascular unit in health and disease. Pharmacol Rev.

[19] Feldman GJ, Mullin JM, Ryan MP (2005). Occludin: structure, function and regulation. Adv Drug Deliv Rev.

[20] Falk G, Fahey T (2009). C-reactive protein and community-acquired pneumonia in ambulatory care: systematic review of diagnostic accuracy studies. Fam Pract.

[21] Harbarth S, Holeckova K, Froidevaux C, Pittet D, Ricou B, Grau GE, Vadas L, Pugin J (2001). Geneva sepsis network: diagnostic value of procalcitonin, interleukin-6, and interleukin-8 in critically ill patients admitted with suspected sepsis. Am J Respir Crit Care Med.

[22] Rochfort KD, Collins LE, McLoughlin A, Cummins PM (2015). Shear-dependent attenuation of cellular ROS levels can suppress proinflammatory cytokine injury to human brain microvascular endothelial barrier properties. J Cereb Blood Flow Metab.

[23] Rodrigues SF, Granger DN (2015). Blood cells and endothelial barrier function. Tissue Barriers.

[24] He H, Geng T, Chen P, Wang M, Hu J, Kang L, Song W, Tang H (2016). NK cells promote neutrophil recruitment in the brain during sepsis-induced neuroinflammation. Sci Rep.

[25] Beringer A, Thiam N, Molle J, Bartosch B, Miossec P (2018). Synergistic effect of interleukin-17 and tumour necrosis factor-α on inflammatory response in hepatocytes through interleukin-6-dependent and independent pathways. Clin Experimental Immunol.

[26] Pan R, Yu K, Weatherwax T, Zheng H, Liu W, Liu KJ (2017). Blood occludin level as a potential biomarker for early blood brain barrier damage following ischemic stroke. Sci Rep.

[27] Ni Y, Teng T, Li R, Simonyi A, Sun GY, Lee JC (2017). TNFα alters occludin and cerebral endothelial permeability: role of p38MAPK. PLoS One.

[28] Qin L-h, Huang W, Mo X-a, Chen Y-l, Wu X-h (2015). LPS induces occludin dysregulation in cerebral microvascular endothelial cells via MAPK signaling and augmenting MMP-2 levels. Oxidative Med Cell Longev.

[29] Zhao Z, Hu J, Gao X, Liang H, Liu Z (2014). Activation of AMPK attenuates lipopolysaccharide-impaired integrity and function of blood–brain barrier in human brain microvascular endothelial cells. Exp Mol Pathol.

[30] Shi S, Qi Z, Ma Q, Pan R, Timmins GS, Zhao Y, Shi W, Zhang Y, Ji X, Liu KJ (2017). Normobaric hyperoxia reduces blood occludin fragments in rats and patients with acute ischemic stroke. Stroke.

[31] Erikson K, Ala-Kokko TI, Koskenkari J, Liisanantti JH, Kamakura R, Herzig KH, Syrjälä H (2019). Elevated serum S-100β in patients with septic shock is associated with delirium. Acta Anaesthesiol Scand.

[32] Abdullah Z, Rakkar K, Bath PMW, Bayraktutan U (2015). Inhibition of TNF-α protects in vitro brain barrier from ischaemic damage. Mol Cell Neurosci.

